# Plasma P-Tau181 for the Discrimination of Alzheimer’s Disease from Other Primary Dementing and/or Movement Disorders

**DOI:** 10.3390/biom12081099

**Published:** 2022-08-10

**Authors:** John S. Tzartos, Fotini Boufidou, Christos Stergiou, Jens Kuhle, Eline Willemse, Lina Palaiodimou, Ioanna Tsantzali, Eleni Sideri, Anastasios Bonakis, Sotirios Giannopoulos, Konstantinos I. Voumvourakis, Georgios Tsivgoulis, Socrates J. Tzartos, Elisabeth Kapaki, George P. Paraskevas

**Affiliations:** 1 2nd Department of Neurology, School of Medicine, National and Kapodistrian University of Athens, “Attikon” General University Hospital, 12462 Athens, Greece; 2 Neurochemistry and Biological Markers Unit, 1st Department of Neurology, “Eginition” Hospital, School of Medicine, National and Kapodistrian University of Athens, 11528 Athens, Greece; 3 Tzartos NeuroDiagnostics, 11523 Athens, Greece; 4 Neurologic Clinic and Policlinic, Departments of Medicine, Clinical Research and Biomedicine, University Hospital Basel, University of Basel, 4031 Basel, Switzerland

**Keywords:** Alzheimer’s disease, dementia, plasma, biomarkers, phospho-tau

## Abstract

Blood phospho-tau181 may offer a useful biomarker for Alzheimer’s disease. However, the use of either serum or plasma phospho-tau181 and their diagnostic value are currently under intense investigation. In a pilot study, we measured both serum and plasma phospho-tau181 (pT181-Tau) by single molecule array (Simoa) in a group of patients with Alzheimer’s disease and a mixed group of patients with other primary dementing and/or movement disorders. Classical cerebrospinal fluid biomarkers were also measured. Plasma (but not serum) pT181-Tau showed a significant increase in Alzheimer’s disease and correlated significantly with cerebrospinal fluid amyloid and pT181-Tau. Receiver operating curve analysis revealed a significant discrimination of Alzheimer’s from non-Alzheimer’s disease patients, with an area under the curve of 0.83 and an excellent sensitivity but a moderate specificity. Plasma pT181-Tau is not an established diagnostic biomarker for Alzheimer’s disease, but it could become one in the future, or it may serve as a screening tool for specific cases of patients or presymptomatic subjects.

## 1. Introduction

Cerebrospinal fluid (CSF) levels of the 3 established (classical) biomarkers for Alzheimer’s disease (AD), namely amyloid peptide β with 42 amino acids (Aβ_42_), tau protein phosphorylated at a threonine residue at position 181 (τ_P-181_) and total tau protein (τ_T_) are the gold standard of fluid-based diagnosis of AD [1,2]. They have been incorporated in diagnostic criteria [3] and they have been considered as core features for the definition of AD as an in vivo biological process, regardless of the stage or the clinical presentation of the disease [4]. However, sampling of CSF requires lumbar puncture (LP). It is a relatively invasive procedure and it may be a source of concern or anxiety for some patients or caregivers. Furthermore, hospitalization may be required in some countries or institutions and the amount of CSF collected is limited.

Blood-based biomarkers have received much attention recently, as a possible diagnostic aid, alternative to CSF biomarkers [5,6]. Blood sampling is an easy-to-perform, non-invasive and acceptable procedure, with no complications, does not require hospitalization and it can be performed in outpatient wards or in the community. It permits the collection of a larger sample volume, suitable for the determination of a wider spectrum of analytes, whilst repeated venipuncture for follow up purposes is far easier and more acceptable than repeated LP. It seems that in AD, changes in plasma levels of classical biomarkers follow similar trajectories as compared to CSF biomarkers, but the magnitude of abnormality in plasma is higher with τ_P-181_ compared to Aβ_42_ or τ_T_ [7]. Indeed, plasma levels of pT181-Tau are 3.5-fold increased in AD as compared to controls [7,8,9,10]; they correlate significantly with CSF levels and parenchymal amyloid and tau load [8] and they may become abnormal in the predementia or even the presymptomatic stage of AD, predicting future transition to AD dementia [10]. Serum levels of τ_P-181_ may also be increased in AD, but they are lower as compared to plasma levels [11]. All those findings may render plasma pT181-Tau a significant screening tool for specific cases, such as: (a) asymptomatic subjects with positive family history of dementia, (b) subjects that do not wish to undergo a lumbar puncture (e.g., patients with subjective cognitive impairment, or patients with doubtful diagnoses), (c) members of the general population, considering the possibility of new or upcoming disease-modifying agents in the future.

In this pilot study, we investigated the levels and diagnostic value of blood pT181-Tau, either serum or plasma, in the real-life necessity to discriminate AD from other dementing and/or movement disorders. 

## 2. Patients and Methods

### 2.1. Patients

A total of 36 patients were included in the study, with no particular selection criteria. They were examined in our department consecutively between October 2020 and October 2021.

The AD group consisted of 12 patients, diagnosed according to the NIA-AA 2018 criteria [4]. They all had mild cognitive impairment or dementia of the amnestic type and, additionally, low CSF Aβ_42_ or Aβ_42_/Aβ_40_ ratio and increased CSF levels of pT181-Tau.

The non-AD group consisted of 24 patients with vascular cognitive impairment (*n*= 11), frontotemporal dementia (*n* = 9), dementia with Lewy bodies (*n* = 3) and multiple system atrophy (*n* = 1), diagnosed according to internationally accepted criteria [12,13,14,15]. None had biomarker levels suggestive of AD [4].

It is noted that no normal control group was included in the present study.

All patients underwent a complete physical and neurological examination as well as neuropsychological testing. The Greek validated version of the Mini Mental State Examination (MMSE) [16], was used as a crude estimate of cognitive dysfunction. Secondary causes of cognitive or movement disorders including thyroid disorders, B12 deficiency, neurosyphilis, brain tumor, subdural hematoma or normal pressure hydrocephalus were excluded. 

A written informed consent was obtained for all cases. The study had the approval of the Bioethics Committee) and the Scientific Board of “Attikon” Hospital (approval numbers 7Γ5/27-7-2021 and 9Γ5/27-7-2021 respectively) and was conducted according to the ethical guidelines of the 1964 Declaration of Helsinki.

### 2.2. Lumbar Puncture and CSF Biomarker Measurements

Lumbar puncture was performed using a standard, 21–22 G, Quincke type needle, at the L4–L5 interspace, between 9 and 12 a.m. according to widely accepted recommendations on standardized operative procedures for CSF biomarkers [17], as described elsewhere [18]. In brief, CSF was collected in 6 polypropylene tubes. The 1st and 2nd tubes (1 mL each) were used for routine CSF cytology and biochemistry. The 3rd tube (2 mL) was used for oligoclonal bands and IgG index determinations. The following 2 tubes (5 mL each) were used for biomarker determinations. The last tube (~2 mL) was used for syphilis serology or other tests according to clinical indications. All CSF samples had <500 red blood cells/μL. The 2 tubes intended for CSF biomarker analysis, were immediately centrifuged (2000× *g* 15 min), aliquoted in polypropylene tubes (1 mL each) and finally stored at −80 °C. Aliquots were thawed only once, just before analysis, which was performed within 6 months of storage. 

Classical CSF biomarkers (Aβ_42_, Aβ_40_, τ_P-181_ and τ_T_) were measured in two laboratories:

(a) In the Neurocheimistry and Biological Markers Unit of the 1st Dept. of Neurology, the CSF biomarkers of 16 patients were determined in a Euroimmun Analyzer I (Euroimmun, Lübeck, Germany), in duplicate, with double sandwich enzyme-linked immunosorbent assay (ELISA) using commercially available kits (EUROIMMUN Beta-Amyloid (1–42) ELISA, EUROIMMUN Beta-Amyloid (1–40) ELISA, EUROIMMUN pTau(181) ELISA and EUROIMMUN Total-Tau ELISA, respectively, Euroimmun, Lübeck, Germany), according to manufacturer’s instructions and by the use of 4-parameter logistic curves, as previously described [18]. Biomarkers were considered normal according to cut-off values of the Unit of Neurochemistry and Biological Markers (Aβ_42_ > 480 pg/mL, Aβ_42_/Aβ_40_ > 0.092, τ_P-181_ < 60 pg/mL, τ_T_ < 400 pg/mL) [18,19].
(b) At Tzartos NeuroDiagnostics, CSF biomarkers of 20 patients were measured by chemilumisence on a Lumipulse 600 G automatic analyzer (Fujirebio, Gent, Belgium), with strict adherence to manufacturer’s instructions. Biomarkers were considered normal according to cut-off values of Tzartos NeuroDiagnostics (Aβ_42_ > 520 pg/mL, Aβ_42_/Aβ_40_ > 0.063, τ_P-181_ < 60 pg/mL, τ_T_ < 360 pg/mL). The cut-off values are comparable with those of other laboratories using the kits for Fujirebio [20].

The CSF AD profile (“fingerprint”) was defined as decreased Aβ_42_ or decreased Aβ_42_/Aβ_40_ and increased τ_P-181_ and thus, compatible with the A^+^T^+^(N)^+^ or A^+^T^+^(N)^−^ profiles of the AT(N) classification system [4].

### 2.3. Blood Sampling and Serum/Plasma Determination of τ_P-181_

Blood was collected after overnight fasting, just prior to the lumbar puncture, in polypropylene tubes (for serum) or polypropylene K3-EDTA tubes (for plasma), according to widely accepted recommendations for sample handling [21,22]. Tubes were centrifuged at 2000× *g* 15 min and the collected serum or EDTA plasma was aliquoted in polypropylene tubes (1 mL each) and stored at −80 °C within 30 min. Frozen serum or plasma aliquots were transferred in dry ice to the Neurologic Clinic and Policlinic, at the University Hospital Basel, Switzerland, for determination of τ_P-181_. For the latter, the method described by Karikari et al. (2020) [11], was performed using the commercially available Simoa (single molecule array) pTau-181 Advantage V2 Kit, on a Simoa HD-X analyzer (Quanterix Corporation, 900 Middlesex Tumpike, Billerica, MA, USA).

### 2.4. Statistical Analysis

All variables were checked for normality and equality of variances by the Shapiro–Wilk’s and Levene’s tests respectively. Demographic and clinical parameters were tested by the χ^2^ test (gender) and *t*-test (numerical variables).

Biomarker levels (except for serum τ_P-181_) deviated significantly from the normal distribution. Logarithmic transformation restored the above violation and permitted the use of parametric tests Since CSF biomarkers were measured in 2 different laboratories with different methods and different reference values, their levels were expressed not by absolute values but by z-scores. Levels of plasma pT181-Tau were first compared by Mann–-Whitney U test and then by 2-way Analysis of Covariance (ANCOVA) with diagnostic group and sex as cofactors and age, disease duration and MMSE score as covariates. T-tests and Pearson correlation coefficients were also used as appropriate. Receiver Operating Characteristics (ROC) curve analysis was used to evaluate the diagnostic value of the various biomarkers.

For statistical analysis the following software packages were used: Statistica version 8.0, 2008 (StatSoft Inc., Tulsa, OK, USA), Prism version 6.01, 2012 (GraphPad Software Inc., San Diego, CA, USA), and MedCalc ^®^ version 12.5 (MedCalc Software, Ostend, Belgium). 

## 3. Results

**Figure 1:**
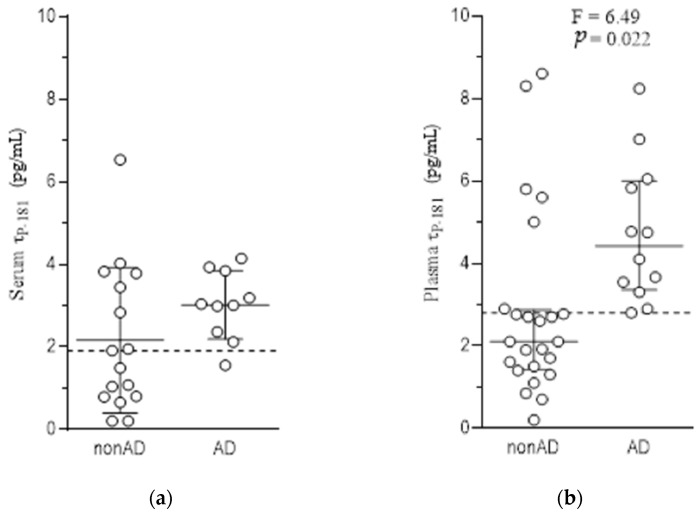
Scatterplots of τ_P-181_ levels in serum (**a**) and plasma (**b**). (**a**) In serum, levels did not deviate significantly from the normal distribution and, thus, bars indicate mean and standard deviation; (**b**) In plasma, they did not distribute normally, thus, bars indicate median and interquartile range. Broken horizontal lines indicate cut-off values suggested by ROC curve analysis. In contrast to serum levels, plasma levels differed significantly in AD, as compared to the non-AD group (2-way-ANCOVA with diagnostic group and sex as cofactors and age, disease duration and MMSE as covariates, after logarithmic transformation of original data).

Results are summarized in Table 1 and Table 2 and Figure 1a,b.

The two diagnostic groups did not differ significantly in respect to sex, age and disease duration, but patients with AD showed lower MMSE scores as compared to the non-AD group. Serum levels of τ_P-181_ were initially measured in a subpopulation of patients (the first 10 with AD and the first 16 with non-AD disorders). No significant difference was noted between the 2 diagnostic groups. Thus, we continued with plasma measurements in the entire population. A significant increase of plasma pT181-Tau was noted in AD, with no effect of age, sex, disease duration or MMSE on the ANCOVA model. Plasma pT181-Tau levels correlated significantly with serum levels and with CSF Aβ and CSF pT181-Tau (Table 3). However, serum pT181-Tau did not correlate significantly with CSF biomarkers.

**Figure 2:**
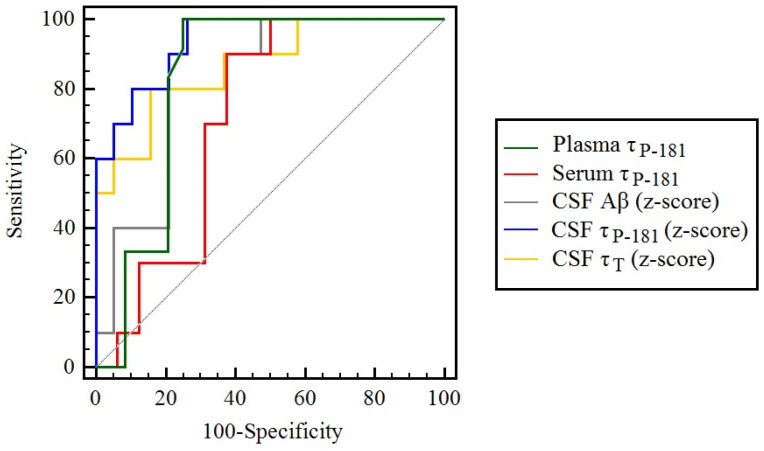
Receiver Operating Characteristics curves of the studied biomarkers. The gray diagonal line indicates a hypothetical curve with an AUC of 0.5.

Analysis of ROC curves revealed statistically significant discrimination between the two studied groups by all biomarkers (Table 4 and Figure 2). No statistically significant difference was noted among the biomarkers (probably as a result of the small number of patients studied), with the notable exception of a superiority of CSF pT181-Tau over serum pT181-Tau. Plasma pT181-Tau showed a very good sensitivity, but a moderate specificity. 

## 4. Discussion

In the present study serum levels of pT181-Tau were lower as compared to the plasma levels. In AD, despite being numerically higher as compared to the non-AD group, no significant result was noted in serum. Furthermore, the serum levels, although correlating significantly with plasma levels, did not correlate significantly with the classical CSF biomarkers (including CSF pT181-Tau). These results do not totally agree with previous studies indicating a good diagnostic performance of serum pT181-Tau or p202-Tau for the discrimination of AD from other dementias or predicting AD progression [23,24,25]. On the contrary, we found that plasma pT181-Tau was significantly increased in AD, as compared to the non-AD group and correlated significantly with CSF Aβ and CSF pT181-Tau. This is in line with recent evidence that, for pT181-Tau, EDTA plasma performs better than serum [26]. A possible restriction for the use of measurements in serum is the preanalytical handling of specimens, and especially the time of incubation needed for clot formation, which may lead to a significant delay in the processing of the samples, whilst, according to guidelines [21,22] the samples should be aliquoted and stored within 60 min or even 30 min. Further research is needed at this point, to clarify the role of additives in preanalytical sampling in different matrices [27].

It has been suggested that plasma levels of pT181-Tau may show a significant diagnostic value, in order to discriminate Alzheimer’s disease from other neurodegenerative disorders and AUCs as high as 0.94–0.98 have been described [10,28]. For the discrimination between AD and FTD, the AUC may be at the level of 0.88 [9]. For the discrimination form vascular dementia, the AUC reaches 0.92, for the discrimination from progressive supranuclear palsy and corticobasal degeneration the AUC reaches 0.88 and, for the discrimination from Parkinson disease or multiple system atrophy, the AUC may reach 0.82, indicating that the diagnostic value of plasma pT181-Tau may approach that of CSF pT181-Tau [11]. In our study, the AUC of plasma pT181-Tau was at the level of 0.83, thus comparable to the previously described values. However, the observed specificity was lower than 80–85%; thus, it was suboptimal according to the 1998 Consensus report of the Working Group on Molecular and Biochemical Markers of Alzheimer’s Disease [29].

The combination with other plasma biomarkers could increase the diagnostic value, but results are conflicting [26,30,31]. Other forms of plasma phospho-tau such as p217-Tau and p231-Tau may perform better than pT181-Tau, especially in early stages of Alzheimer’s disease, but much work has to be done in this direction [31,32,33]. Perhaps the optimization of methodologies used for the measurement and a combination of multiple phospho-tau isoforms could help increase the specificity for AD. [34] However, given the existing sensitivity of the biomarker, it can serve as an adequate screening tool in cases such as people with subjective cognitive impairment or prodromal AD. In previous studies it has also been shown to present a correlation with patients’ cognitive scores and hippocampal atrophy [35].

Limitations of the present study were the small number of patients included and the absence of a normal control group. However, this was a pilot study, aiming to investigate the diagnostic potential in AD vs. non-AD disorders. Studying a larger number of AD and non-AD patients in different and larger groups, including the frontotemporal lobar degenerations, various synucleinopathies, various subtypes of vascular cognitive impairment and various types of controls (normal, psychiatric) is mandatory.

## 5. Conclusions

CSF biomarkers still remain the gold standard for “fluid-based diagnosis” of AD. Plasma pT181-Tau is not yet an established biomarker, but it may become so in the future. The results of the present pilot study are encouraging and support the study of EDTA plasma pT181-Tau (and not serum) as a possible biomarker of AD. Additionally, they support the performance of larger scale studies, in order to optimize the diagnostic potential of plasma pT181-Tau.

## Figures and Tables

**Table 1:** Clinical and biochemical data of the subjects (serum subpopulation).

	Non-AD	AD	*p* Value
Gender (males/females)	16 (7/9)	10 (5/5)	NS ^1^
Age (years)	67.0 ± 12.3	68.5 ± 16.1	NS ^2^
Disease Duration (years)	2.96 ± 1.27	3.88 ± 2.50	NS ^2^
MMSE	23.3 ± 8.50	15.5 ± 8.80	0.034 ^2^
Serum τ_P-181_ (pg/mL)	2.16 ± 1.77	3.01 ± 0.83	NS ^2^
CSF Aβ (z-score)	−0.49 ± 1.52	−1.63 ± 1.21	0.056 ^2^
CSF τ_P-181_ (z-score)	0.11 ± 0.92	3.51 ± 2.48	<0.0001 ^2^
CSF τ_Τ_ (z-score)	0.25 ± 1.12	2.53 ± 2.47	0.004 ^2^

Results are presented as mean ± standard deviation. AD: Alzheimer’s Disease, MMSE: Mini Mental State Examination, CSF: Cerebrospinal fluid, Aβ: either Aβ_42_/Aβ_40_ or Aβ_42_ alone (when Aβ_40_ was not available), τ_P-181_: tau protein phosphorylated at a threonine residue at position 181, τ_Τ_: total tau protein, NS: non-significant. ^1^ χ^2^-test, ^2^ *t*-test.

**Table 2:** Clinical and biochemical data of the subjects (plasma, entire population).

	Non-AD	AD	*p* Value
Gender (males/females)	24 (13/11)	12 (6/6)	NS ^1^
Age (years)	69.6 ± 7.51	68.7 ± 15.8	NS ^2^
Disease Duration (years)	3.06 ± 2.50	3.60 ± 2.21	NS ^2^
MMSE	26.3 ± 2.35	16.4 ± 8.20	<0.001 ^2^
Plasma τ_P-181_ (pg/mL)	2.84 ± 2.23	4.75 ± 1.72	0.022 ^3^
	2.1 (1.43–2.87)	4.42 (3.36–5.99)	0.009 ^4^
CSF Aβ (z-score)	−0.05 ± 1.42	−1.71 ± 0.95	0.003 ^2^
CSF τ_P-181_ (z-score)	0.22 ± 1.13	3.58 ± 2.23	<0.001 ^2^
CSF τ_Τ_ (z-score)	0.22 ± 1.18	3.29 ± 2.79	0.007 ^2^

Results are presented as mean ± standard deviation. For plasma which follows the log-normal distribution, median values (25th–75th percentiles are also shown) AD: Alzheimer’s Disease, MMSE: Mini Mental State Examination, CSF: Cerebrospinal fluid, Aβ: either Aβ_42_/Aβ_40_ or Aβ_42_ alone (when Aβ_40_ was not available), τ_P-181_: tau protein phosphorylated at a threonine residue at position 181, τ_Τ_: total tau protein, NS: non-significant. ^1^ χ^2^-test, ^2^ *t*-test, ^3^ 2-way-ANCOVA with diagnostic group and sex as cofactors and age, disease duration and MMSE as covariates (after logarithmic transformation of original data), ^4^ Mann–Whitney U test.

**Table 3:** Correlation of serum and plasma levels of pT181-Tau with the other biomarkers.

	Serum τ_P-181_ (pg/mL)	Plasma τ_P-181_ (pg/mL)
Plasma τ_P-181_ (pg/mL)	0.85 (<0.001)	-
CSF Aβ (z-score)	−0.23 (NS)	−0.59 (<0.01)
CSF τ_P-181_ (z-score)	0.15 (NS)	0.40 (0.05)
CSF τ_Τ_ (z-score)	0.05 (NS)	0.25 (NS)

Results are presented as Pearson correlation coefficient (*p* value, Bonferroni corrected). CSF: Cerebrospinal fluid, Aβ: either Aβ_42_/Aβ_40_ or Aβ_42_ alone (when Aβ_40_ was not available), τ_P-181_: tau protein phosphorylated at a threonine residue at position 181, τ_Τ_: total tau protein, NS: non-significant. ^1^ χ^2^-test, ^2^ *t*-test.

**Table 4:** Receiver Operating Characteristics (ROC) analysis of the discriminant value for each of the studied biomarkers.

Biomarker	AUC	Cut-Off Value	Sensitivity	Specificity	*p* Value
Plasma τ_P-181_ (pg/mL)	0.83	2.80	1.00	0.75	<0.0001
	(0.67–0.93)		(0.74–1.00)	(0.53–0.90)	
Serum τ_P-181_ (pg/mL)	0.72	1.94	0.90	0.63	0.034
	(0.51–0.88)		(0.56–0.98)	(0.35–0.85)	
CSF Aβ (z-score)	0.82	–1.27	0.80	0.79	0.0001
	(0.63–0.93)		(0.44–0.98)	(0.54–0.94)	
CSF τ_P-181_ (z-score)	0.94 *	0.77	1.00	0.74	<0.0001
	(0.78–0.99)		(0.69–1.00)	(0.49–0.91)	
CSF τ_T_ (z-score)	0.87	0.98	0.80	0.84	<0.0001
	(0.69–0.96)		(0.44–0.98)	(0.60–0.97)	

AUC: Area under the ROC curve. *p* values indicate the level of significance as compared to an AUC of 0.5. Values in parentheses indicate the 95% confidence interval. * *p* = 0.04 vs. serum τ_P-181_.

## Data Availability

The data presented in this study are available on request from the corresponding author. The data are not publicly available due to privacy restrictions.
